# Chemerin enhances the adhesion and migration of human endothelial progenitor cells and increases lipid accumulation in mice with atherosclerosis

**DOI:** 10.1186/s12944-020-01378-5

**Published:** 2020-09-20

**Authors:** Jue Jia, Fan Yu, Yuyun Xiong, Weiping Wei, Hong Ma, Fulvio Nisi, Xu Song, Ling Yang, Dong Wang, Guoyue Yuan, Hongwen Zhou

**Affiliations:** 1 Department of Endocrinology and Metabolism, the First Affiliated Hospital of Nanjing Medical University, 300 Guangzhou Road, Nanjing, Jiangsu China; 2 Department of Emergency, the Affiliated Hospital of Jiangsu University, Zhenjiang, Jiangsu China; 3 Department of Endocrinology, the Affiliated Hospital of Jiangsu University, Zhenjiang, Jiangsu China; 4 Department of Clinical Laboratory, the Affiliated Hospital of Jiangsu University, Zhenjiang, Jiangsu China; 5 Department of Dermatology, the Affiliated Hospital of Jiangsu University, Zhenjiang, Jiangsu China; 6 Department of Anesthesiology, Intensive Care and Pain Therapy Centre, Hospital Santa Maria della Misericordia, Perugia, Italy

**Keywords:** Lipid metabolism, Adipokines, Chemerin, Endothelial progenitor cells (EPCs), Atherosclerosis, Plaque, Inflammation, MAPK pathway

## Abstract

**Background:**

The role of adipokines in the development of atherosclerosis (AS) has received increasing attention in recent years. This study aimed to explore the effects of chemerin on the functions of human endothelial progenitor cells (EPCs) and to investigate its role in lipid accumulation in ApoE-knockout (ApoE−/−) mice.

**Methods:**

EPCs were cultured and treated with chemerin together with the specific p38 mitogen-activated protein kinase (MAPK) inhibitor SB 203580 in a time- and dose-dependent manner. Changes in migration, adhesion, proliferation and the apoptosis rate of EPCs were detected. ApoE−/− mice with high-fat diet-induced AS were treated with chemerin with or without SB 203580. Weights were recorded, lipid indicators were detected, and tissues sections were stained.

**Results:**

The data showed that chemerin enhanced the adhesion and migration abilities of EPCs, and reduced the apoptosis ratio and that this effect might be mediated through the p38 MAPK pathway. Additionally, chemerin increased the instability of plaques. Compared with the control group and the inhibitor group, ApoE−/− mice treated with chemerin protein had more serious arterial stenosis, higher lipid contents in plaques and decreased collagen. Lipid accumulation in the liver and kidney and inflammation in the hepatic portal area were enhanced by treatment with chemerin, and the size of adipocytes also increased after chemerin treatment. In conclusion, chemerin can enhance the adhesion and migration abilities of human EPCs and reduce the apoptosis ratio. In animals, chemerin can increase lipid accumulation in atherosclerotic plaques and exacerbate plaques instability. At the same time, chemerin can cause abnormal lipid accumulation in the livers and kidneys of model animals. After specifically blocking the p38 MAPK pathway, the effect of chemerin was reduced.

**Conclusions:**

In conclusion, this study showed that chemerin enhances the adhesion and migration abilities of EPCs and increases the instability of plaques and abnormal lipid accumulation in ApoE−/− mice. Furthermore, these effects might be mediated through the p38 MAPK pathway.

## Background

Atherosclerosis (AS) is the pathological basis of most cardiovascular and cerebrovascular diseases and can cause a high burden of cardiovascular diseases [1]. However, the exact mechanism of AS development has not yet been clarified. Several studies have indicated that lipid metabolism dysfunction and inflammation dominate in the pathological process of AS and that the interaction between these processes promotes AS progression [1–3]. The formation of atherosclerotic plaques is also closely related to lipid disorders and inflammatory responses. Under the combined action of various cellular and molecular events, the evolution of AS is a continuous process in which early fatty lesions are converted to highly vulnerable fragile plaques [4]. Adipocytokines secreted by adipocytes play a critical role in lipid metabolism and inflammation, which are emerging targets in AS and related diseases [5–7]. Representative adipocytokines, such as hypersensitive C-reactive protein (hsCRP), leptin, adiponectin and tumour necrosis factor-α (TNF-α), participate in AS by affecting the brain, the liver, skeletal muscle, islets, gonads, immune organs and the vasculature in a systematic manner [8–10].

Chemerin is an adipocytokine involved in the recruitment of immune cells [11] and serves as a bridge between various immune cells from the initial stage of inflammatory diseases through the development process [12], and its receptor CMKLR1 (also named ChemR23 in humans) is a specific functional member of the G protein receptor family. Chemerin exerts opposing anti-inflammatory and pro-inflammatory effects at different stages of the inflammatory response via the chemerin/CMKLR1 pathway [13, 14]. Clinical studies have found that chemerin is tightly associated with metabolic syndrome and related diseases through the regulation of glucose and lipid metabolism. Significantly higher chemerin concentrations are found with obesity than with normal weight, and chemerin is significantly related to body mass index and waist circumference [15–17]. Recent studies have also concluded that circulating serum chemerin levels are positively correlated with the numbers of unstable plaques and diseased vessels [18, 19]. In human endothelial cells, chemerin can activate major pathways that regulate angiogenesis, including the mitogen-activated protein kinase (MAPK) and protein kinase B (AKT) pathways. In addition, Georgios K. Dimitriadis et al. found that chemerin can affect the adhesion between monocytes and endothelium, thus inducing endothelial cell inflammation, and this interesting finding provides novel insights into chemerin and AS [20].

Endothelial progenitor cells (EPCs) are classic cell models of AS due to their potential to differentiate into vascular endothelial cells in the circulating peripheral blood [21]. Disruption of the balance between EPC-mediated damage and repair is the crucial initiating parameter in AS. However, whether chemerin can affect the functions of EPCs and how chemerin participates in the development of atherosclerotic plaques in vivo are unclear.

Therefore, in this study, EPCs and ApoE-knockout (ApoE−/−) mice were utilized to explore the effect of chemerin under the background of AS.

## Methods

### Isolation and culture of EPCs

Human peripheral blood mononuclear cells (MNCs) were isolated from healthy adult peripheral venous blood via density gradient centrifugation, resuspended in endothelial cell growth medium-2 (EGM-2) (Lonza, Walkersville, MD USA, catalogue #: CC-4147), and plated in human fibronectin (HFN, Beijing Solarbio Science & Technology Co., Ltd., Beijing, China, catalogue #: CH38) -coated plates at a density of 2–5 × 10^6^ cells/well. The culture medium was changed for the first time on the 4th day and then changed every 2 days thereafter.

### Identification of EPCs

To identify EPCs, the cells were stained with antibodies against the endothelial markers CD34, CD14, CD133 (eBioscience, California, USA, catalogue #: 11–0349, 15–0149, 12–1338) and VEGFR-2 (BD, New Jersey, United States, catalogue #: 560495), and flow cytometry analysis was performed. Dual staining was used to observe acetylated low-density lipoprotein (Dil-ac-LDL, Beijing Solarbio Science & Technology Co., Ltd., Beijing, China, catalogue #: H7970) uptake and fluorescein isothiocyanate (FITC)-labelled *Ulex europaeus* Agglutinin I (UEA-I-FITC, Sigma, Missouri, USA, catalogue #: L9006) binding.

### Treatment of EPCs

After culturing for 7 days, the adherent cells were resuspended and randomly divided into the control group, recombinant human chemerin protein (R&D California, USA, catalogue #: 2324-CM-025/CF) treatment groups and inhibitor group (SB 203580, Sigma, Missouri, USA, catalogue #: S8307). For the dose-dependent study, the chemerin treatment groups included 2.5 ng/mL, 25 ng/mL, 50 ng/mL and 100 ng/mL protein-stimulated groups. The inhibitor group was stimulated with 50 ng/mL chemerin protein plus 10 μmol/L SB 203580. EPCs were treated for 24 h. For the time-dependent study, cells were stimulated with chemerin protein at 50 ng/mL for 0 h, 12 h, 24 h, 36 h and 48 h.

### Cell adhesion assay

The cells were harvested and resuspended and their concentration was adjusted to 2 × 10^6^ cells/mL. The cells were plated into a 96-well plate (200 μL/well) that had been coated with HFN for 30 min. Then, the plate was placed in an incubator (37 °C, 5% CO_2_, 95% humidity) for 30 min. Adherent cells were counted in three randomly selected fields.

### Cell proliferation assay

After cell culture and stimulation were completed, the cells were resuspended and adjusted to a concentration of 2 × 10^6^ cells/mL. The cell suspensions were inoculated into 96-well plates coated with HFN, and every sample was replicated three times. Ten microliters of sterile MTT (5 mg/mL, Sigma, Missouri, USA, catalogue #: M-2128) was added to each well. After incubation for 4 h, the supernatant was discarded, and 150 μL of DMSO (Sigma, Missouri, USA, catalogue #: D2650) was added to each well. The plate was shaken for 10 min, and the absorbance value was measured at a detection wavelength of 490 nm.

### Apoptosis assay

The cells in each group described above were collected after stimulation was completed and stained using an apoptosis detection kit (BD, New Jersey, United States, catalogue #: 556547). Cells were washed twice with cold PBS and resuspended at a concentration of 1 × 10^6^ cells/mL. Then, the solution was transferred to a culture tube, and Annexin V and propidium iodide (PI) were added. After incubation for 15 min at room temperature in the dark, the cells were analysed via flow cytometry within 1 h.

### Migration assay

After the cells were cultured for 7 days, the cells were harvested and resuspended in medium without FBS, and the cell concentration was adjusted to 5 × 10^5^ cells/mL. Complete medium containing recombinant human VEGF165 (100 ng/mL, Peprotech, New Jersey, USA, catalogue #: 100–20) was added to the lower chamber. After 24 h of culture, cells were counted and photographed in 5 random fields.

### Animal models

All specific-pathogen-free (SPF) clean-grade mice were purchased from Changzhou Cavens Lab Animal Co. (Changzhou, China) and raised at the laboratory animal research centre of Jiangsu University. six- to eight-week-old male C57BL/6 J mice were set as the wild-type (WT) group (*n* = 10, WT) and fed on regular food until the end of the experiment, and 6- to 8-week-old male ApoE−/− mice were set as the experimental groups. WT mice were fed a classic high-fat diet for 16 weeks and randomly divided into 3 groups: the control group (*n* = 6, control), chemerin protein group (*n* = 8, chemerin), and inhibitor group (*n* = 8, inhibitor). Drugs were given according to mouse body weight starting at the 8th week (recombinant mouse chemerin protein, R&D California USA, catalogue #: aa 17–156, 50 ng/(kg × d) i.p. for 4 weeks or chemerin together with the specific p38 MAPK-specific inhibitor SB 203580 (SB 203580, Selleck, Texas, USA, catalogue #: S1076, 10 mg/(kg × d) i.p. for 4 weeks) Mouse body weights were measured every two weeks after the drug interventions.

### Animal samples

All mice were anaesthetized after fasting for 12 h, and blood was collected and centrifuged to obtain serum after standing at room temperature. Tissue samples were stored at − 80 °C for later use. The aorta was separated from the root of the vein to the bifurcation of the arteria iliaca communis. The arteries, aortic root, liver, kidney and white adipose tissue (WAT) were fixed with 4% paraformaldehyde for later staining.

### Detection of the lipid index

Liver lipids were extracted from equal masses of liver tissue using absolute ethanol as a medium. The serum and liver concentrations of cholesterol (T-CHO), triglyceride (TG), high-density lipoprotein cholesterol (HDL) and low-density lipoprotein cholesterol (LDL) were measured with enzyme-linked immunosorbent assays (Nanjing Jiancheng Bioengineering Institute, China, catalogue #: A111–1-1, A110–1-1, A112–1-1 and A113–1-1).

### Tissue staining

Whole arteries were subjected to Oil red O staining. The aortic roots of mice were sectioned at the same plane, and the sections were subjected to haematoxylin-eosin (HE) staining and Movat staining. The liver sections were subjected to HE staining and Oil red O staining. The kidney sections and WAT sections were subjected to HE staining. Image-Pro Plus software (Media Cybernetics, Inc., Rockville, MD, USA) was used to analyse images of the aortic roots, liver, kidney and WAT. The number of adipocytes was determined using AdipoCount software (http://www.csbio.sjtu.edu.cn/bioinf/AdipoCount/).

### Statistical analysis

All experiments involving cell culture studies were repeated three times with three replicates per experiment. Shapiro-Wilk tests were used to determine the normality of the data. The data are presented as the means ± standard deviation (SD) or as the median (range) as appropriate. Statistical comparisons among groups were performed using one-way analysis of variance (ANOVA), *t-*tests and nonparametric tests as appropriate. *P < 0.05* was considered statistically significant.

## Results

### Identification of EPCs

**Fig. 1:**
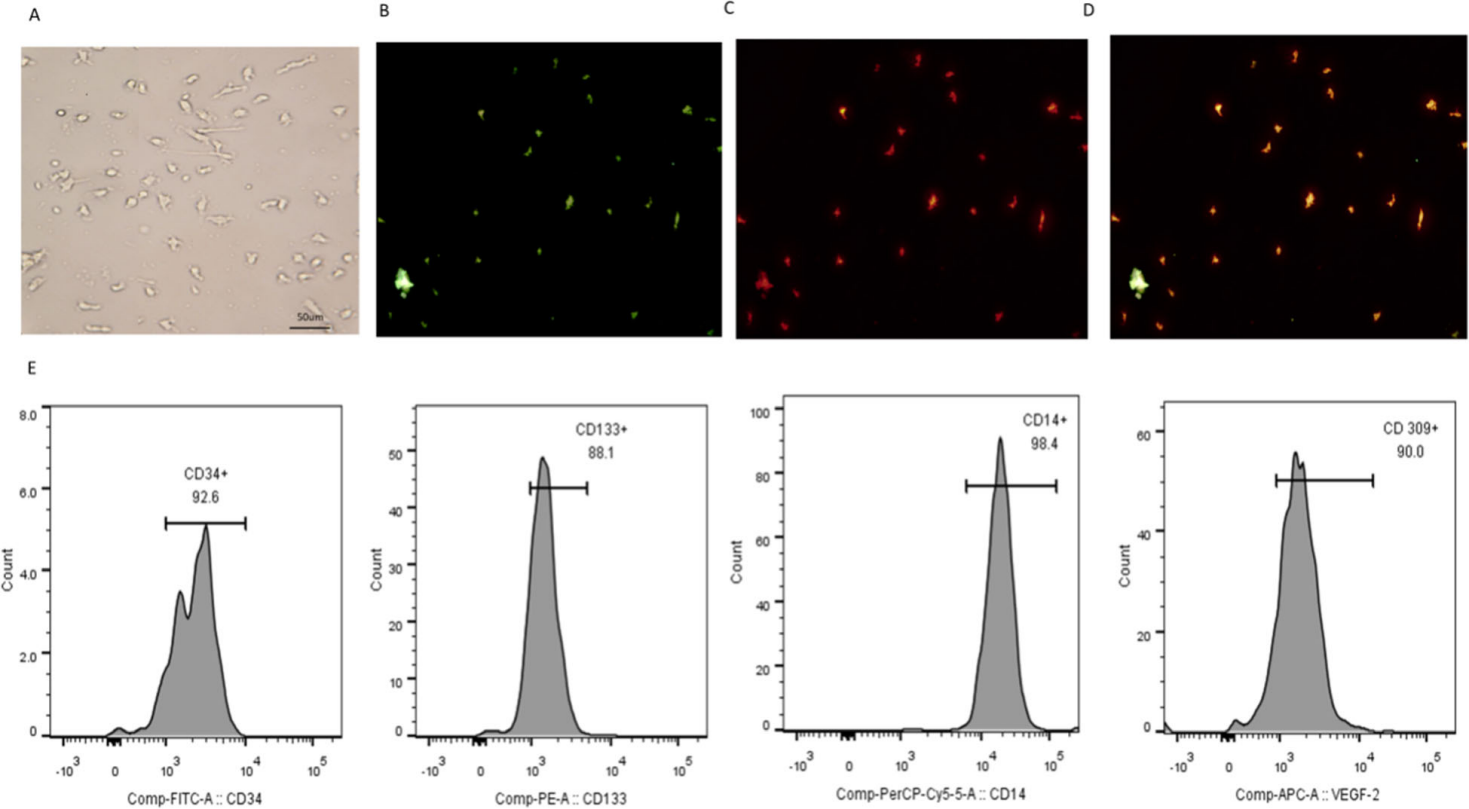
Identification of EPCs. **a** Adherent cells grew in a blood island manner. Fluorescent staining of EPCs. **b** Adherent cells took up UEA-1-lectin. **c** Adherent cells took up Dil-Ac-LDL. **d** Adherent cells took up UEA-1-lectin and Dil-Ac-LDL. E. Surface molecular markers of EPCs. Adherent cells expressed CD34, CD133, CD14 and VEGFR-2. All experiments involving cell culture studies were repeated three times with three replicates per experiment

On the seventh day of cell culture, cell morphology was observed. The cells grew in clusters with short rod-like shapes and formed colonies similar to a blood island morphology (Fig. 1 a). EPCs were also characterized as adherent cells that were double-positive for Dil-Ac-LDL uptake and FITC-UEA-1 lectin binding (87.7% ± 15.2%) (Fig. 1 b, c and d). Flow cytometry results showed that the expression levels of the EPC surface markers CD133, CD14, CD34 and VEGF-R2, as surface markers for EPCs, were 91.1 ± 3.7, 96.8 ± 2.3, 95 ± 3.0 and 95.3% ± 4.7%, respectively (Fig. 1 e).

### Effect of chemerin on the functions of EPCs

**Fig. 2:**
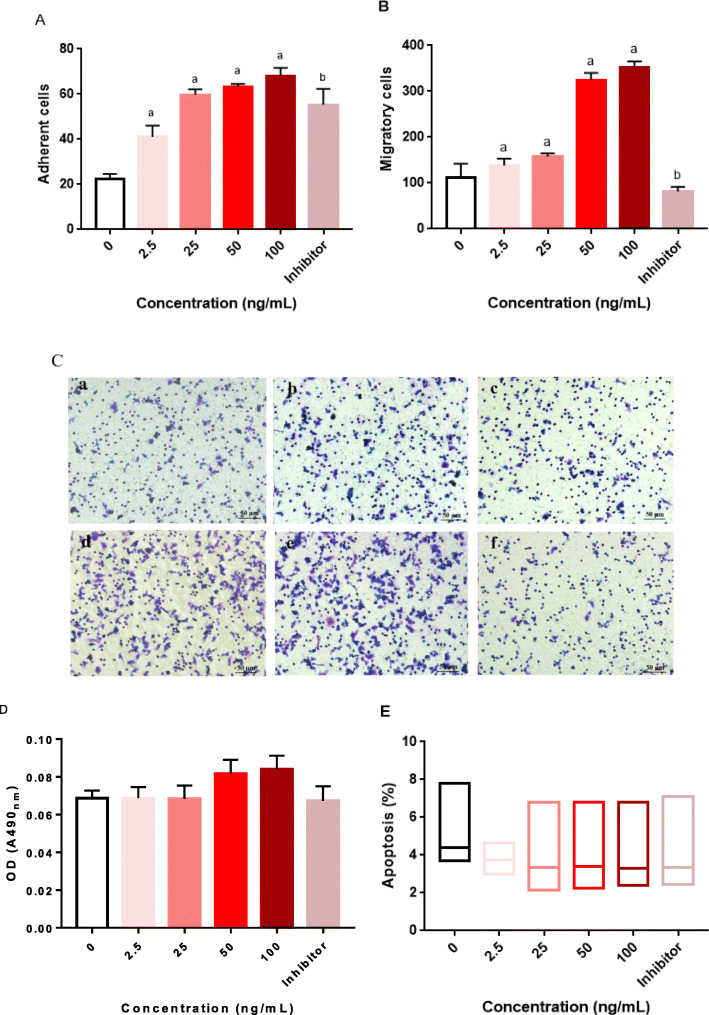
Dose-dependent effects of chemerin on the biological characteristic of EPCs. The control group was not subjected to any drug treatment; the control group received only the same volume of solvent used for the drug treatments in the treatment groups. The chemerin treatment groups included 2.5 ng/mL, 25 ng/mL, 50 ng/mL and 100 ng/mL protein-stimulated groups. EPCs were treated for 24 h. The inhibitor group was stimulated with 50 ng/mL chemerin protein plus 10 μmol/mL SB 203580. **a** Adherence of EPCs, a: VS. the control group, *P < 0.05*; b: the 50 ng/mL group VS. the inhibitor group, *P < 0.05*. The data are presented as the means ± SD. **b** Migration of EPCs, a: VS. the control group, *P < 0.05*; b: the 50 ng/mL group VS. the inhibitor group, *P < 0.05*. The data are presented as the means ± SD. **c** Image of EPC migration, a: the control group, b: the 2.5 ng/mL group, c: the 25 ng/mL group, d: the 50 ng/mL group, e: the 100 ng/mL group and f: the inhibitor group. **d** Proliferation of EPCs. The data are presented as the means ± SD. **e** Apoptosis ratio of EPCs. The data are presented as the median (range). All experiments involving cell culture studies were repeated three times, with three replicates per experiment

**Fig. 3:**
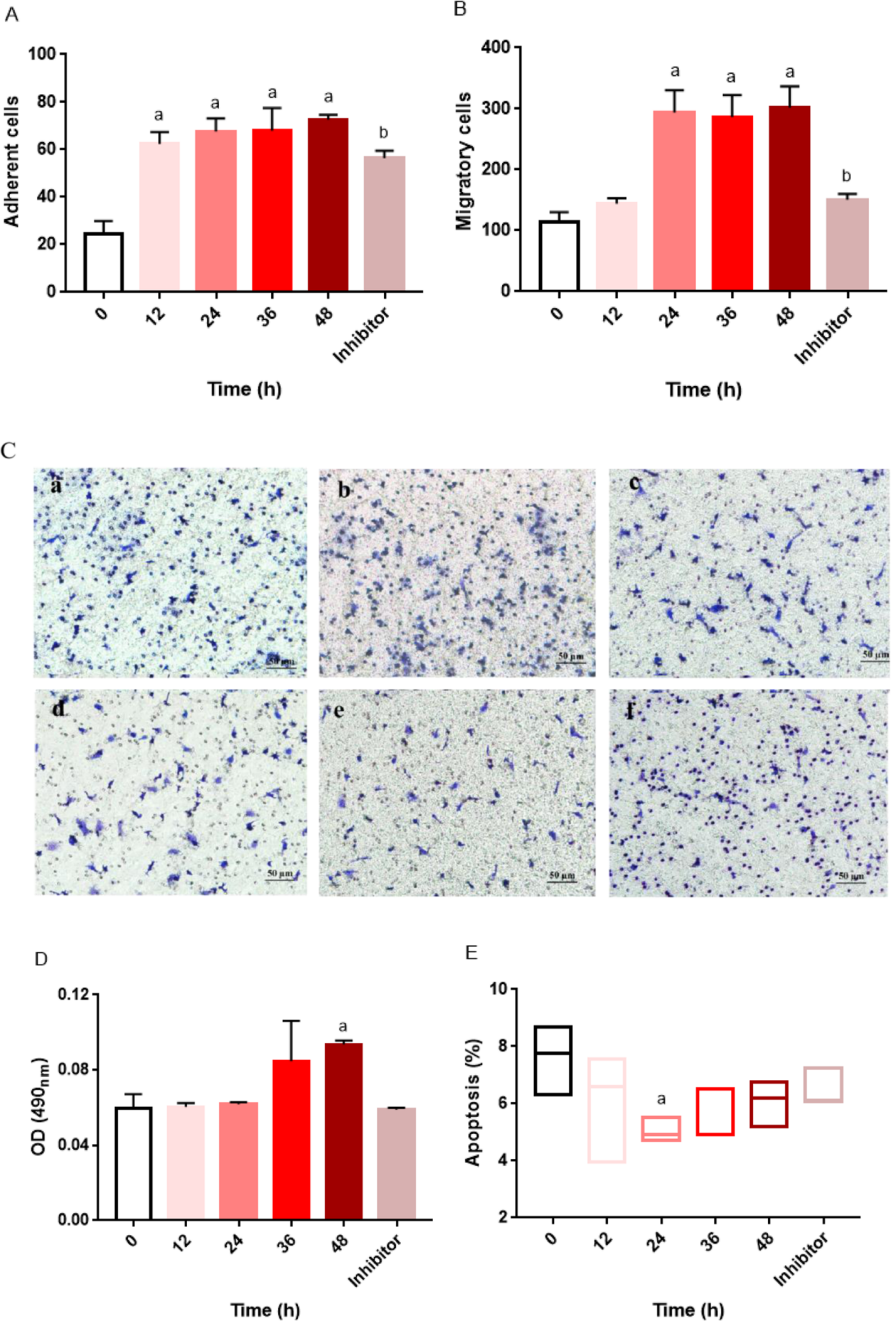
Time-dependent effects of chemerin on the biological characteristic of EPCs. The control group was not subjected to any drug treatment; the control group received only the same volume of solvent used for the drug treatments in the treatment groups. The chemerin treatment groups included 12 h, 24 h, 36 h and 48 h protein-stimulated groups. EPCs were treated with chemerin at a concentration of 50 ng/mL. The inhibitor group was stimulated with 50 ng/mL chemerin protein plus 10 μmol/mL SB 203580 for 24 h. **a** Adherence of EPCs, a: VS. the control group, *P < 0.05*; b: the 24 h group VS. the inhibitor group, *P < 0.05*. The data are presented as the means ± SD. **b** Migration of EPCs, a: VS. the control group, *P < 0.05*; b: the 24 h group VS. the inhibitor group, *P < 0.05*. The data are presented as the means ± SD. **c** Image of EPC migration, a: the control group, b: the 12 h group, c: the 24 h group, d: the 36 h group, e: the 48 h group and f: the inhibitor group. **d** Proliferation of EPCs. The data are presented as the means ± SD. **e** Apoptosis ratio of EPCs. The data are presented as the median (range). All experiments involving cell culture studies were repeated three times, with three replicates per experiment

As shown in Fig. 2 a, b, and c, the adherence and migration capacities of EPCs were enhanced in a dose-dependent manner when EPCs were treated with chemerin. A specific inhibitor of the p38 MAPK pathway alleviated this chemerin-induced effect. The proliferation of EPCs increased in line with the increase in chemerin concentration (Fig. 2 d), but only the difference between the 100 ng/mL chemerin protein-stimulated group and the control group was statistically significant (*P <* 0.05). As the concentration of the stimulating protein increased, the apoptosis ratio gradually decreased. However, the result was not statistically significant (Fig. 2 e). Furthermore, with extension of the stimulation time, the adherence and migration capacities of EPCs were also enhanced (Fig. 3 a, b and c). In addition, the number of EPCs attached to the wall also increased significantly in a time-dependent manner (Fig. 3a). A specific inhibitor of the p38 MAPK pathway alleviated this chemerin-induced effect. The proliferation of EPCs increased in line with the increase in stimulation time (Fig. 3 d), but no changes were evident until the stimulation time reached 48 h. In addition, the apoptosis ratio significantly decreased after stimulation for 24 h (Fig. 3 e).

### Effect of chemerin on the body weights of ApoE−/− mice

Eight weeks after the start of the drug intervention, the body weights of the mice in each group were measured every 2 week. The weight value in each group increased gradually but without significant differences between the groups (Table 1).

**Table 1:** Effects of chemerin on weight changes in different groups

Body weight (g)	0 week	2nd week	4th week	6th week	8th week
WT group	28.89 ± 1.86	29.40 ± 1.78	30.24 ± 1.86	31.25 ± 1.74	31.54 ± 1.95
Control group	29.05 ± 1.34	29.22 ± 1.57	30.08 ± 1.97	31.15 ± 2.07	31.93 ± 1.73
Chemerin group	29.16 ± 2.09	29.12 ± 1.17	30.50 ± 1.95	31.73 ± 2.07	32.20 ± 1.91
Inhibitor group	29.06 ± 1.41	28.9 ± 1.12	29.37 ± 1.17	29.94 ± 1.34	30.13 ± 1.49

The weights of all mice were recorded after the start of the drug intervention. The results are expressed as the mean ± SD. The WT group (*n* = 10, WT) was fed regular food until the end of the experiment, and ApoE−/− mice were fed a classic high-fat diet for 16 weeks and were randomly divided into 3 groups: the control group (*n* = 6, control), the chemerin protein group (*n* = 8, chemerin), and the inhibitor group (*n* = 8, inhibitor). Drugs or the same volume of solvent were given according to mouse body weight (recombinant mouse chemerin protein, 50 ng/(kg × d) i.p. for 4 weeks) or chemerin together with the p38 MAPK-specific inhibitor SB203580 (SB 203580; 10 mg/(kg × d) i.p. for 4 weeks) starting at the 8th week.

### Effects of chemerin on lipid parameters in ApoE−/− mice

**Fig. 4:**
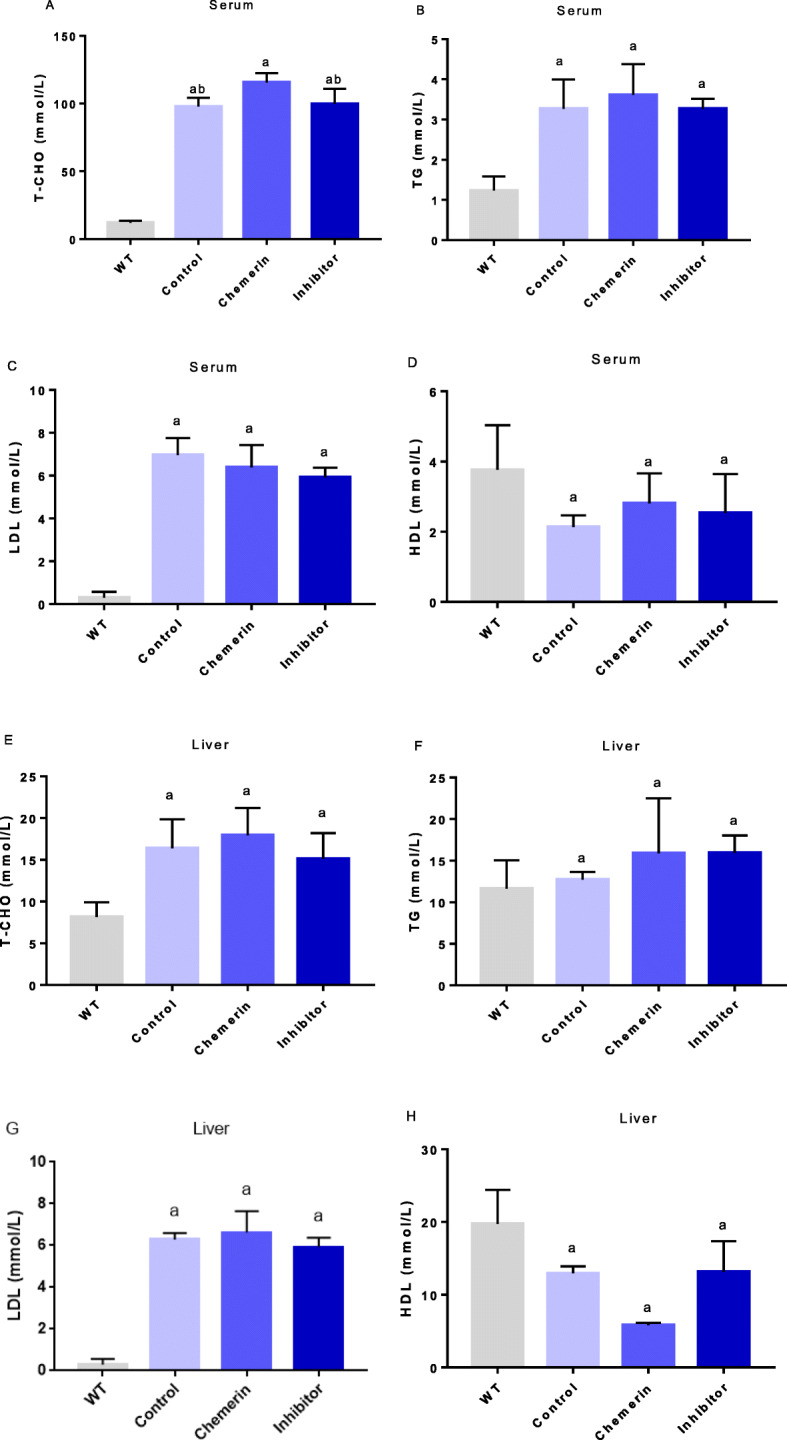
Effects of chemerin on lipid parameters in ApoE−/− mice. WT mice were not treated with any drugs (*n* = 10). The control group (*n* = 6) was injected with PBS by weight, the chemerin group (*n* = 8) was injected with chemerin protein by weight, and the inhibitor group (*n* = 8) was injected with chemerin protein and the p38 MAPK-specific inhibitor SB 203580 by weight. The results are presented as the mean ± SD. **a** Serum T-CHO concentration. The results are expressed as the mean ± SEM. **b** Serum TG concentration. **c** Serum LDL concentration. **d** Serum HDL concentration. **e** Liver T-CHO concentration. **f** Liver TG concentration. **g** Liver LDL concentration. **h** Liver HDL concentration. a: VS. the WT group, *P < 0.01*; b: VS. the chemerin group, *P < 0.01*

Compared with that in WT mice, the serum lipid concentration in all ApoE−/− mice was significantly increased, and among lipids, the T-CHO concentration was higher in the chemerin group than in the control group. However, this effect was reversed after administration of the p38 MAPK inhibitor, and the difference was statistically significant (Fig. 4 a). In addition, no significant difference in the concentrations of TG, LDL and HDL were found between each ApoE−/− group (Fig. 4 b, c and d). The liver lipid concentrations showed a trend similar to that of the serum concentrations (Fig. 4 e, f, g and h).

### Effect of chemerin on atherosclerotic plaques in ApoE−/− mice

**Fig. 5:**
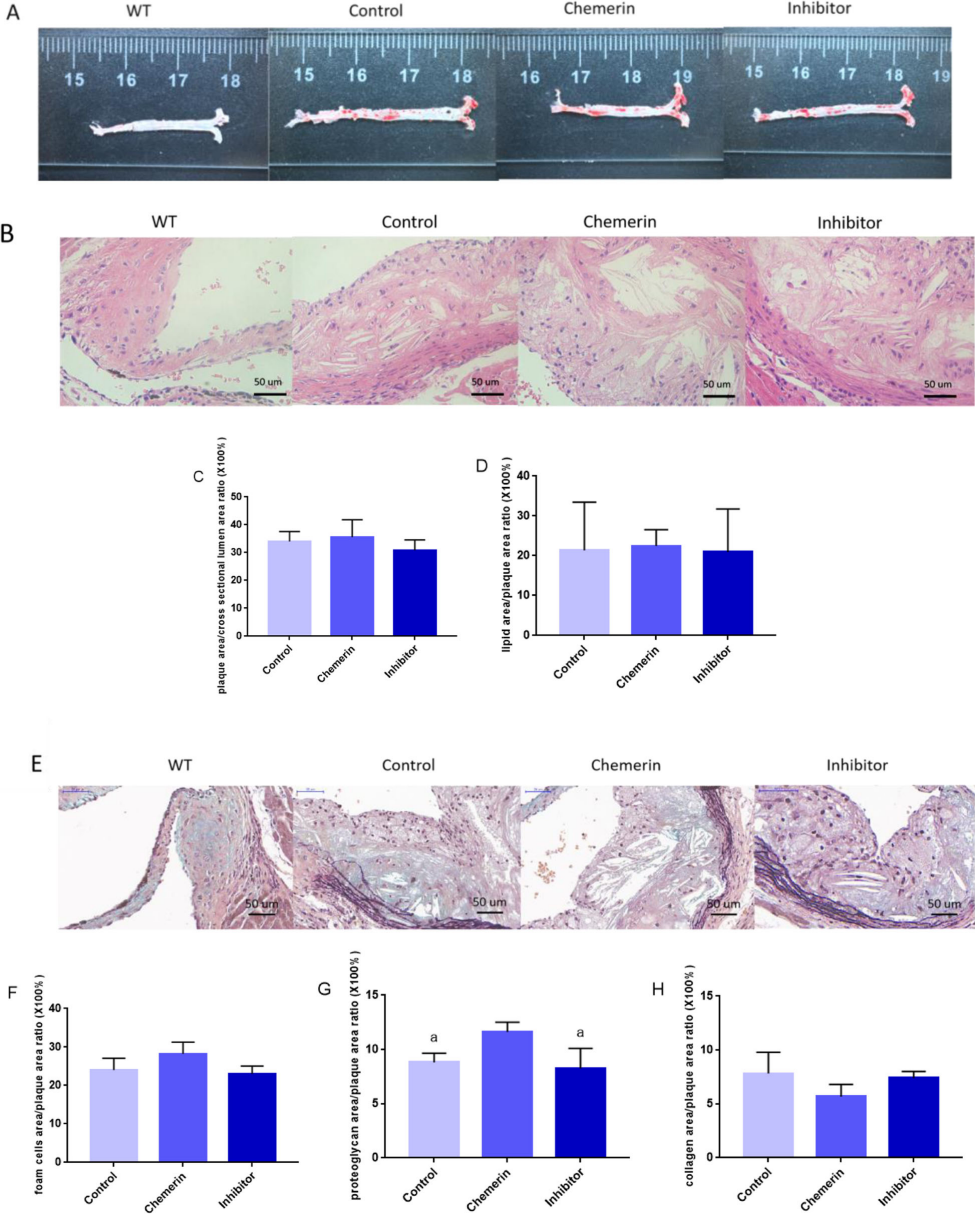
Effect of chemerin on atherosclerotic plaques in ApoE−/− mice. **a** Oil red O staining of the aorta. **b** Aortic root sections in different ApoE−/− mouse groups after HE staining. **c** The ratio of the plaque area to the cross-section of the vessel lumen area (Control group, *n* = 6; Chemerin group, *n* = 8; Inhibitor group, *n* = 8). The results are presented as the mean ± SD. **d** The ratio of the lipid area to the plaque cross-sectional area (Control group, *n* = 6; Chemerin group, *n* = 8; Inhibitor group, *n* = 8). The results are presented as the mean ± SD. **e** Aortic root sections from different ApoE−/− mouse groups after Movat staining. **f** The ratio of foam cell to plaque areas (Control group, *n* = 6; Chemerin group, *n* = 8; Inhibitor group, *n* = 8). The results are presented as the mean ± SD. **g** The ratio of the proteoglycan area to the plaque area (Control group, *n* = 6; Chemerin group, *n* = 8; Inhibitor group, *n* = 8). a: VS. the chemerin group, *P < 0.05*. The results are presented as the mean ± SD. **h** The ratio of the collagen area to the plaque area. The results are presented as the mean ± SD

Oil red O staining of aortas and the lipid index results verified that the AS models were built successfully (Fig. 5 a). HE staining showed that cholesterol crystals were formed in the plaques of all ApoE−/− mice (Fig. 5 b). The ratio of plaque cross-sectional area to the cross-sectional area of the vessel lumen area and the ratio of the lipid area to the plaque cross-sectional area were larger in the chemerin protein-treated group than in the control group, and the effect was attenuated after blocking the p38 MAPK pathway (Fig. 5 c, d). Movat staining showed that foam cells appeared in all ApoE−/− mice (Fig. 5 e). In the chemerin group, the ratios of the foam cell area and proteoglycan area to the plaque area were larger (Fig. 5 f, g) than in control group, and SB 203580 weakened the effect. Significant differences in the proteoglycan area/plaque area ratio (× 100%) were identified among the three groups. Correspondingly, the ratio of the collagen content in the plaque was decreased in the chemerin group compared with the ratio in the control group, while in the inhibitor group, the collagen levels were slightly increased compared with the levels in the chemerin-treated group (Fig. 5 h).

### Effects of chemerin on the liver, kidney and WAT in ApoE−/− mice

**Fig. 6:**
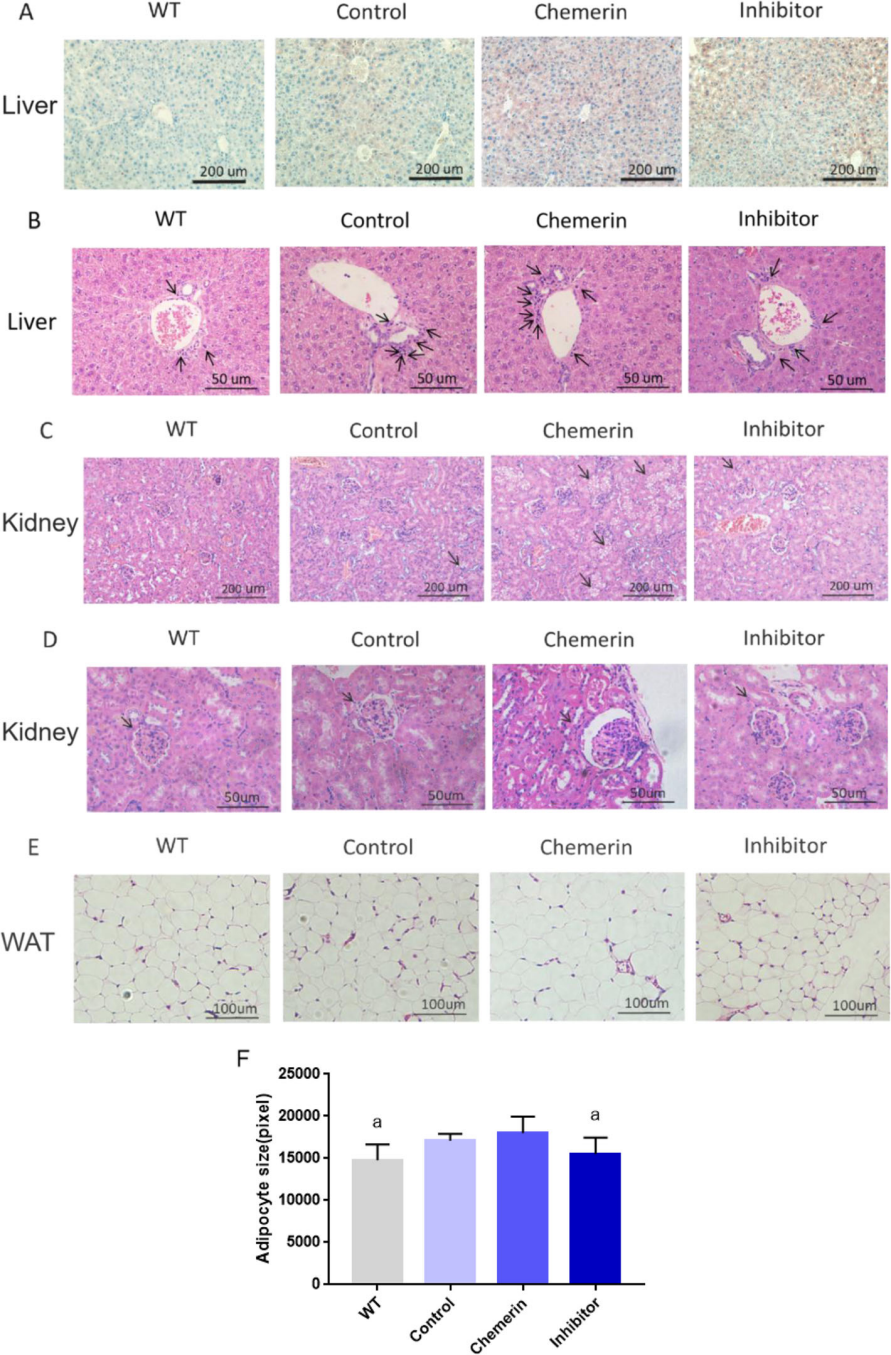
Effects of chemerin on the liver and kidney in ApoE−/− mice. **a** Oil red O staining of the liver in the four groups. **b** HE staining of the liver portal area. The arrows indicate inflammatory cells. **c** HE staining of the kidney. The arrows indicate vacuoles in the cytoplasm. **d** HE staining of the kidney. The arrows indicate glomeruli. **e** HE staining of WAT. **f** The sizes of adipocytes in WAT (WT group, *n* = 10; Control group, *n* = 6; Chemerin group, *n* = 8; Inhibitor group, *n* = 8). The results are presented as the mean ± SD. a: VS. the chemerin group, *P < 0.05*

The results of liver Oil red O staining showed that chemerin protein could increase lipid accumulation in the liver, and this effect was reduced after blocking the p38 MAPK pathway (Fig. 6 a); these lipid metabolism results were in line with the serum and liver lipid concentration results. The inflammatory cell number was increased in the hepatic portal area under stimulation with chemerin, and this acceleration phenomenon was reduced with the use of SB 203580 (Fig. 6 b). In addition, in HE-stained renal tissue, lipid accumulation increased with chemerin treatment, while this phenomenon diminished after blocking of the p38 MAPK pathway (Fig. 6 c). The glomerulus was found to be atrophied and hardened after the protein intervention, and the tubules were swollen and congested (Fig. 6 d). In addition, compared with that in WT mice, the size of adipocytes was increased in the WAT from chemerin-treated ApoE−/−mice, but this effect was weakened by p38 MAPK pathway inhibition (Fig. 6 e, f).

## Discussion

Recently, the endothelial injury response theory in AS pathogenesis has gained support from most scholars [22]. The main risk factors for AS were believed to cause the inflammatory fibrosis of the endothelium and intima, eventually damaging the arteries. Many studies have been performed to investigate the role of adipokines in AS. The results have shown that adipokines can participate in AS by affecting glucose metabolism, lipid metabolism and vascular function [23]. Chemerin, which is considered an adipokine, was first identified in 2007. Recently, chemerin has been shown to be closely related to inflammation, metabolic syndrome and cardiovascular diseases [24]. Several studies have concluded that chemerin can be considered a marker of a high atherosclerotic risk [25, 26]; however, the specific mechanism of chemerin in AS development has not yet been clarified.

AS develops from endothelial cell damage, and the role of EPCs in vascular repair presents new research targets in the mechanism of AS. In this study, the results showed that chemerin enhanced the adhesion of EPCs in a dose- and time-dependent manner. In addition, the proliferation of EPCs increased with chemerin in a dose-dependent manner. Previous studies have shown that chemerin plays an important role in cancer development due to its promotion of the formation of new blood vessels in tumour tissue [27], and EPCs are closely related to neovascularization, which is consistent with the research results. In the present study, the migration ability of EPCs gradually increased with the increase in chemerin concentrations and the stimulation time. Furthermore, chemerin decreased the apoptosis ratio of EPCs. A study by Goralski KB et al. found that chemerin can enhance the adhesion and migration of endothelial cells [28]. EPCs are the precursor cells of endothelial cells, which mainly exist in the bone marrow. Both endothelial progenitor cells and endothelial cells belong to endothelial lineage cells, and they can express the same specific surface markers. And EPCs could exhibit endothelial cell attributes after specific culture and induction [29]. Yuan Kong et al. showed that the p38 MAPK pathway downregulation and the Akt pathway upregulation induced by atorvastatin could improve the quantity and function of EPCs from bone marrow in corticosteroid-resistant ITP patients [30]. Similar to previous findings, the present study showed that chemerin had a promotive effect on EPCs. Additionally, the results of this study showed that the apoptosis ratio of EPCs decreased only under short-term stimulation, which might be due to an effect on feedback regulation. In addition, this research found that after blocking the p38 MAPK pathway, the effect of chemerin on EPCs was weakened, indicating that chemerin might regulate EPCs via the p38 MAPK pathway. An earlier study showed that p38 MAPK is an important pathway involved in the regulation of intracellular and extracellular immune inflammation and blood vessel formation [31], which is consistent with this study. Previous studies have showed that chemerin could induce both MAPK and AKT phosphorylation in endothelial cells [32]. Meanwhile, chemerin has been shown to activate p38 MAPK and AKT phosphorylation in human placental microvascular endothelial cells [33]. Another study showed that the number and function of EPCs could be regulated via the p38 MAPK pathway and the AKT pathway [30]. After blocking the PI3K/AKT pathway, the monocyte-endothelial adhesion induced by chemerin was significantly improved as well as the attenuation of NF-κB activation [20]. In the current research, results showed that chemerin could participate in the modulation of EPCs via p38 MAPK pathway. Therefore, we speculated that the effects of chemerin on EPCs might also be regulated by blocking the AKT pathway.

Previous evidence has shown that a variety of physiological responses, including innate and adaptive immune responses, are involved in the development and progression of AS [34, 35]. The vulnerability of AS plaques is related to the variety of cells involved in the formation of AS and the corresponding cytokines. Chemerin could bind to its specific receptors that expressed in several immune cells such as monocytes, T cells, natural killer (NK) cells and dendritic cells (DC) to participate in immune response. Downregulation of chemerin receptor could promote the activation of NK cells only during short-term intervention [36]. Additionally, the chemerin level is positively related to percentages of Th9 and Th17 in asthma patients [37]. Meanwhile, the expression of chemerin was higher in breast cancer tissue that is contrast to the results showed in acute myeloid leukemia patients [36]. Therefore, although the results of in vitro experiments showed that chemerin promotes EPCs, the effect of chemerin on AS is still unclear.

Based on the results of the in vitro study, the role of chemerin in vivo was further studied. In the in vivo study, plaque distributions were investigated throughout the aorta in all ApoE−/− mice. Staining of plaques from ApoE−/− mice showed that chemerin increased the lipid content in the plaques and reduced the collagen content, thus increasing the possibility of plaque rupture. In a previous clinical study, chemerin levels were elevated in patients with coronary heart disease and were associated with the number of unstable plaques and the number of coronary lesions [24, 38], and the results of this study are in accordance with those findings. In this study, all lipid parameters in ApoE−/− mice were significantly higher than those in WT mice, which is consistent with the genetic characteristics of the model animals. The serum concentration of T-CHO was significantly higher after treatment with chemerin, and this effect was reversed when the p38 MAPK pathway inhibitor was used. However, TG, LDL and HDL levels did not change significantly. Among them, the serum HDL concentration presented an upward trend under treatment with chemerin, which may have been be due to changes in the composition and function of HDL in a disease state. An earlier study found that the HDL component in patients with coronary heart disease differs from that in healthy controls [39]. An increased level of inflammatory HDL can lead to changes in the HDL phenotype, leading to an increase in LDL oxidation, a vascular inflammatory response and impaired cholesterol reverse transport.

Chemerin can affect multiple organs due to the wide distribution of its specific receptors [11]. The expression levels of liver chemerin have been found to be independently and positively related to liver steatosis, lobular inflammation, balloon dilation and fibrosis [40]. In this study, chemerin increased lipid accumulation in both the liver and kidney. The levels of liver lipids in ApoE−/− mice were higher than those in WT mice. After chemerin intervention, fatty infiltration increased and was accompanied by increased inflammatory infiltration in the portal area, which is consistent with previous work [40]. In the kidney, an increased number of vacuoles was observed in the tubules and glomeruli, indicating increased lipid accumulation in the kidney. Studies have shown a clear link between circulating chemerin levels and renal function, and chemerin levels appear to increase as renal function declines [41]. Disruption of circulating lipids not only increases the production of reactive oxygen species in the kidney but also stimulates the production of pro-inflammatory factors, and this process may cause a pathological change similar to AS in the kidney. Disorder of cellular cholesterol in the kidney can hinder the outflow of cholesterol, which may increase abnormal lipid accumulation and foam cell, formation and ultimately lead to a malicious behavioural cycle. As an adipokine secreted by adipose tissue, chemerin can also act on adipose tissue. The present study showed that the size of adipocytes in WAT became larger under chemerin intervention, although no difference in mouse weights in each group were observed, which might have been due to the relationship between chemerin and fat distribution. In addition, studies have shown that adipocytokines secreted by adipose tissue in different regions may have different effects on inflammation [42]. WAT can secrete more pro-inflammatory adipocytokines and enhance inflammation in the body [43], and this adipokine- imbalance is closely related to the development of AS [44]. Therefore, chemerin might aggravate the disorder and the development of the inflammatory response on the basis of AS lesions.

### Study strengths and limitations

In this study, the effect of chemerin protein on EPCs was first explored and its influences on the liver, kidney and WAT were further studied. The adhesion and migration of EPCs were enhanced by chemerin, and this effect might be mediated through the p38 MAPK pathway. These results provide further mechanistic insight into the action of chemerin in the development of AS. However, some limitations of this study should be disclosed. Because the EPCs were obtained entirely from healthy volunteers, studies on EPCs derived from cardiovascular patients were not conducted. In addition, after vascular injury, EPCs in peripheral blood mobilized by the bone marrow are recruited to the lesion [45], and thus, studies related to intramedullary EPCs in mice after long-term chemerin intervention also need to be carried out. Additionally, an in vivo study should be performed to investigate changes in the expression of specific proteins and inflammatory factors related to the p38 MAPK pathway and some other pathways such as AKT and ERK pathway in blood vessels. Considering the interaction between chemerin and inflammatory response as well as the close relationship of AS and inflammation, the number and composition ratio of immun cells as well as the quantity of EPCs might change after chemerin intervention in ApoE−/− mice, this exploration also deserved to conduct. In addition, it has been previously demonstrated that inhibition of the p38 MAPK pathway alone can ameliorate AS [46], and thus, whether the effects observed in inhibitor group mice can be attributed to blocking of the interaction between chemerin and the p38 MAPK pathway needs to be explored. Furthermore, relevant exploration of AS development in chemerin-knockdown mice should also be explored in the future.

## Conclusion

In conclusion, this study showed that chemerin can enhance the adhesion and migration abilities of EPCs and increase the instability of plaque and abnormal lipid accumulation in ApoE−/− mice. Moreover, these effects might be mediated through the p38 MAPK pathway. This research may provide insight into the relationship between the adipokine chemerin and AS, contribute to further exploration of the exact mechanism of AS and broaden the treatment of AS.

## Data Availability

The datasets used and/or analysed in the current study are available from the corresponding author upon reasonable request.

## Abbreviations

AS: Atherosclerosis
TNF-α: Tumour necrosis factor-α
MAPK: Mitogen-activated protein kinase
AKT: Protein kinase B
EPCs: Endothelial progenitor cells
MNCs: Mononuclear cells
T-CHO: Total cholesterol
TG: Triglyceride
HDL: High-density lipoprotein cholesterol
LDL: Low-density lipoprotein cholesterol
HE: Haematoxylin-eosin
